# MRI detection of endothelial cell inflammation using targeted superparamagnetic particles of iron oxide (SPIO)

**DOI:** 10.1186/s40169-016-0134-1

**Published:** 2017-01-02

**Authors:** Joyce M. S. Chan, Maggie S. H. Cheung, Richard G. J. Gibbs, Kishore K. Bhakoo

**Affiliations:** 1 Department of Surgery, Prince of Wales Hospital, The Chinese University of Hong Kong, Hong Kong SAR, People’s Republic of China; 2 Regional Vascular Unit, St Mary’s Hospital, Imperial College Healthcare NHS Trust, Imperial College London, London, UK; 3 Translational Molecular Imaging Group, Singapore Bioimaging Consortium, Agency for Science, Technology and Research (A*STAR), 11 Biopolis Way, Helios, 138667 Singapore

**Keywords:** Endothelial adhesion molecules, Atherosclerosis, MRI, Superparamagnetic particles of iron oxide, Vascular imaging, Inflammation imaging

## Abstract

**Background:**

There is currently no clinical imaging technique available to assess the degree of inflammation associated with atherosclerotic plaques. This study aims to develop targeted superparamagnetic particles of iron oxide (SPIO) as a magnetic resonance imaging (MRI) probe for detecting inflamed endothelial cells.

**Methods:**

The in vitro study consists of the characterisation and detection of inflammatory markers on activated endothelial cells by immunocytochemistry and MRI using biotinylated anti-P-selectin and anti-VCAM-1 (vascular cell adhesion molecule 1) antibody and streptavidin conjugated SPIO.

**Results:**

Established an in vitro cellular model of endothelial inflammation induced with TNF-α (tumor necrosis factor alpha). Inflammation of endothelial cells was confirmed with both immunocytochemistry and MRI. These results revealed both a temporal and dose dependent expression of the inflammatory markers, P-selectin and VCAM-1, on exposure to TNF-α.

**Conclusion:**

This study has demonstrated the development of an in vitro model to characterise and detect inflamed endothelial cells by immunocytochemistry and MRI. This will allow the future development of contrast agents and protocols for imaging vascular inflammation in atherosclerosis. This work may form the basis for a translational study to provide clinicians with a novel tool for the in vivo assessment of atherosclerosis.

## Background

Atherosclerosis is a dynamic, progressive disease arising from the combination of endothelial dysfunction with a compensatory inflammatory response [1]. Inflammation is not only instrumental in the development of atheromatous plaques, but is also a key driver of plaque instability subsequently developing clinically significant symptoms such as stroke and myocardial infarction [1].

Current clinical techniques for imaging atherosclerotic disease include angiography, magnetic resonance angiography (MRA), computed tomography angiography (CTA) or ultrasound which principally gives an accurate assessment of degree of luminal stenosis caused by atheroma. However, none of these techniques offer insight about the biological behaviour of atheromatous plaques—particularly degree of inflammation—which cause significant clinical events such as plaque instability and rupture [2].

MRI (magnetic resonance imaging) has advantages over other imaging modalities in clinical use, as it is non-invasive, does not involve ionising radiation and is able to provide high resolution images of the vessel wall at a sub-millimetre level [3]. In addition, recent development of advanced MR contrast agents that selectively target molecules involved in plaque inflammation, offering the promise of in vivo molecular MR imaging of atherosclerosis [4] and ex vivo MR imaging of vulnerable carotid plaque [5].

By exploiting specific ‘inducible’ molecular targets, or cellular events in diseases, molecular imaging uses targeted imaging agents to generate the image contrast [6]. One of the earliest events in atherosclerosis is the over-expression of adhesion molecules on the activated endothelium following exposure to inflammatory cytokines [7]. These pathophysiologically ‘inducible’ endothelial adhesion molecules, such as VCAM-1 (vascular cell adhesion molecule-1) and P-selectin, have the potential to serve as attractive biomarkers for imaging inflammation in atherosclerosis. VCAM-1 (CD106) is an immunoglobulin superfamily glycoprotein (100–110 kDa) expressed on activated endothelial cells, macrophages and smooth muscle cells [8]. It has a vital role in monocyte recruitment in early stages of atherosclerosis [9]. Its expression is upregulated by activated endothelium under inflammation, unlike resting endothelial cells [8]. The increased level of expression appears to be correlated with the extent of exposure to the atherosclerotic risk factors [8]. P-selectin (CD62P, GMP-140, PADGEM), a single-chain glycoprotein, is an adhesion molecule expressed on the surface of activated endothelial cells, which line the luminal surface of blood vessels, and on activated platelets [10]. It mediates initial leukocyte rolling, preceding leukocyte diapedesis into the atherosclerotic lesions [10, 11]. Similar to VCAM-1, P-selectin is rapidly mobilized to the surface of endothelial cells and platelets in response to stimuli [10]. These features render VCAM-1 and P-selectin an ideal biomarker for functional molecular imaging and targeted therapeutics in early atherosclerosis.

To achieve targeted molecular MR imaging, smarter contrast agents are required to identify the molecules of interest (i.e. endothelial adhesion molecules in atherosclerosis) with high specificity. Specificity can be achieved through conjugation of contrast agents with monoclonal antibodies, or their immunospecific fragments F(ab) or peptides. Recent advances in this area include oxidized low-density lipoprotein (oxLDL)-targeted iron oxide nanoparticles [12] and scavenger receptor-AI-targeted iron oxide nanoparticles [13].

SPIO (superparamagnetic particles of iron oxide) nanoparticles are composed of an iron oxide core coated with a biocompatible polymer with size ranging from 50–300 nm. Various iron-oxide nanoparticle preparations are under clinical trials and their safety profile has been increasingly recognised [14]. Iron-oxide particles have become the favoured contrast agent for a number of reasons. Firstly, they have a known biocompatibility profile with their degradation occurring through normal physiologic iron metabolism pathways [15]. By contrast, gadolinium chelates, the clinically used MRI contrast agent, has been reported to have potential severe long-term toxicity effects, including nephrogenic sclerosing fibrosis in patients with impaired renal function [16]. Secondly, iron-oxide particles have high sensitivity due to iron-oxide’s inherent superparamagnetism. They distort magnetic fields, creating marked contrast effects far exceeding their physical size, thereby strongly enhancing the transverse relaxation times T2 and T2*. Hence, they act as contrast agents for T2 sequence [15]. T2 technique is a relaxation time measurement contributing to the transverse decay of the MR signal that arise from natural interactions at the atomic and molecular levels within the tissue or substance of interest. T2* can be considered an “observed” T2, whereas T2 can be considered the “true” T2 of the tissue being imaged [17].

Iron-oxide nanoparticles have the potential to open up new avenues into molecular MR imaging, in both pre-clinical and clinical studies. Indirect quantitative methods utilising the non-specific uptake of iron-oxide particles by macrophages to identify plaque inflammation, have been reported [18]. The present study is an exploratory qualitative study aimed at visualising and characterising atherosclerosis using targeted SPIO as an MRI probe for detecting inflamed endothelial cells. It is hypothesized that highly specific antibody-conjugated SPIO would enable MRI of inflamed endothelial cells.

## Methods

### Induction of inflammation on endothelial cells by TNF-α

Mouse aortic endothelial cells (mAEC) (Innoprot, Spain) were cultured in an endothelial cell medium consisting of Dulbecco’s Modified Eagle Medium containing 4500 mg/L glucose, l-glutamine and pyruvate, supplemented with 25 μg/mL of gentamicin, 2 mM glutamine, 5% of fetal calf serum, 1 μg/mL of hydrocortisone, 10 units of heparin, 10 ng/mL of recombinant human epidermal growth factor (PeproTech Ltd, London, UK) and 3 ng/mL of recombinant human fibroblast growth factor (PeproTech Ltd, London, UK). mAEC were subsequently incubated with 0.1, 1, 10 and 100 ng/mL recombinant mouse TNF-α (Sigma, MO, USA) for 4, 24 and 48 h at 37 °C to induce endothelial VCAM-1 and P-selectin expression. mAECs without exposure to TNF-α served as controls.

### Detection of inflammatory markers on mAEC by immunocytochemistry

mAECs on coverslips were harvested and fixed with 4% paraformaldehyde (PFA) for 15 min, permeabilized with 0.1% Triton X-100 for 5 min and blocked with 5% bovine serum albumin (BSA) for 30 min. Cells were then incubated with biotinylated primary antibody against VCAM-1 (Vector Laboratories, Burlingame, USA) and P-selectin (Vector Laboratories, Burlingame, USA) at room temperature for 1 h, followed by streptavidin antibodies (Alexa Fluor^®^ 594 streptavidin, Thermofisher Scientific, Inc.) for 30 min at room temperature. After repeated wash with PBS, coverslips were mounted on a slide in antifade reagent with DAPI (ThermoFisher Scientific Inc.). An Olympus IX-83 inverted microscope fitted with a 60×, 1.4 NA oil immersion objective (Olympus), and a monochrome CCD camera (Olympus) driven using CellSens (Olympus) were used. VCAM-1 and P-selectin fluorescence intensity per cell were quantified using software ImageJ [19, 20]

$$\begin{aligned} &{\text{Corrected total cell fluorescence}} \; ( {\text{CTCF}} ) \\& = {\text{Integrated Density}} - ( {\text{Area of selected cell}} \times {\text{Mean fluorescence of background readings}} ) \end{aligned}$$

### Detection of inflammatory markers on mAEC by MRI

**Fig. 1:**
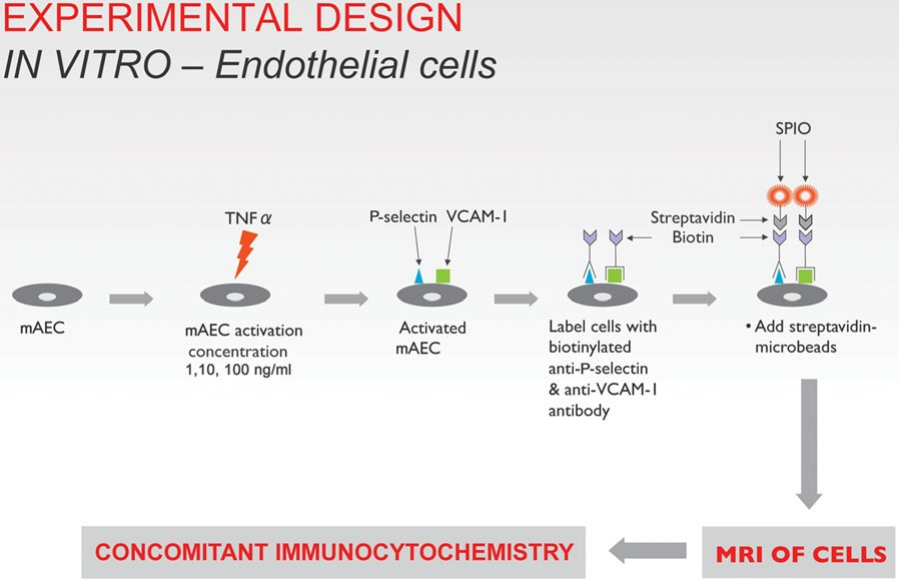
Detection and characterisation of P-selectin and VCAM-1 on activated endothelial cells by antibody-conjugated SPIO

mAECs on the culture plates were used for the SPIO targeting to biotinylated antibodies conjugated on the cell surface as illustrated in Fig. 1. Firstly, the activated cells (1, 10 and 100 ng/mL TNF-α, 4 h) were washed twice by centrifugation (5000 rpm for 5 min) and incubated at room temperature for 20 min with the biotinylated anti-P-Selectin and anti-VCAM-1 at a concentration of 10 μg/mL diluted in 10% rabbit serum for labelling 10^7^ cells. Following incubation, the cells were washed twice to remove unbound biotinylated antibodies with 2 mL of labelling buffer composed of PBS (1×), supplemented with 2 mM EDTA, and centrifuged. The buffer was kept at 4 °C throughout the experiment to prevent capping of antibodies on the cell surface and non-specific cell labelling. The endothelial cell pellet was re-suspended in 90 μL of labelling buffer per 10^7^ cells and 10 μL of streptavidin microbeads (Miltenyi Biotec Ltd, Surrey, UK) per 10^7^ cells. These streptavidin microbeads are iron-oxide beads (SPIO of 50 nm diameter) conjugated to streptavidin and can be used as targeted ‘contrast agents’ for activated endothelial cells, thereby making these cells ‘MR visible’. The cells were then incubated with the beads at 4 °C for 15 min and washed to remove unbound streptavidin microbeads afterwards. The cells were finally re-suspended in 150 μL of PBS and 150 μL of 2% agarose for cell phantom MRI. Inactivated cells (without TNF-α exposure) and activated cells incubated with biotinylated anti-mouse IgG (Vector Laboratories, Burlingame, USA) served as controls.

**Fig. 2:**
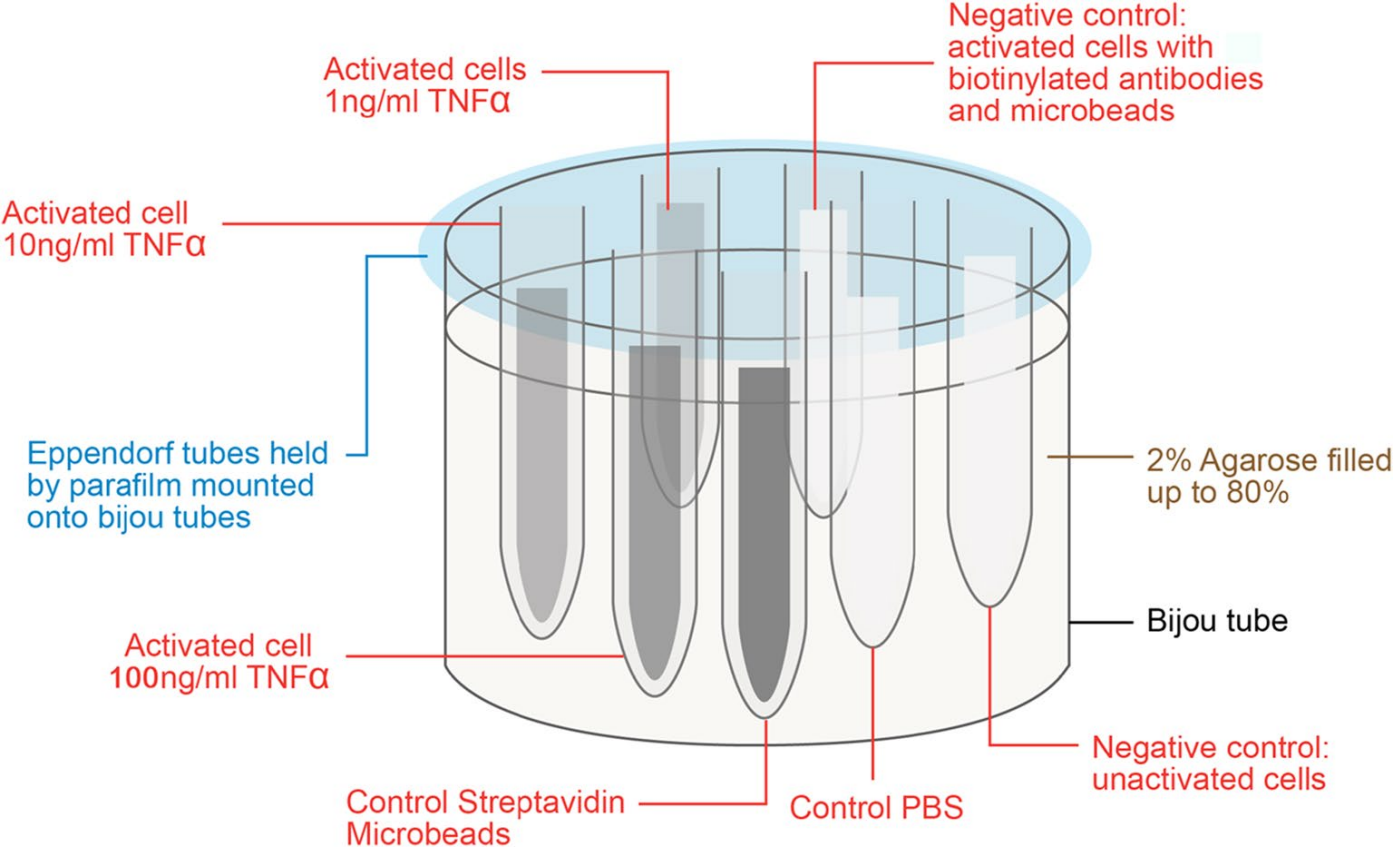
Cell phantom preparation. Six samples were prepared. They are the activated cells stimulated by TNF-α of different concentration (1 ng/mL, 10 and 100 ng/mL). The controls are activated cells with biotinylated IgG antibodies and microbeads as negative control, unactivated cells as negative control, and streptavidin microbeads

The cell suspensions in the tubes were embedded in 2% agarose block (Sigma^®^, UK, Fig. 2). The cell phantom MRI was performed using a three tesla clinical whole body scanner (Achieva TX, Philips Healthcare, Best Netherlands). The samples were placed in a receive-only eight-channel SENSE wrist coil for optimal signal reception. A T2-weighted sequence (TR 2000 ms; TE 120 ms; number of slices 10; slice thickness 1 mm; FOV 80 mm; signal averages 30; matrix 266 × 336) was employed in the transverse plane to obtain sectional images of the samples. The MR images were analysed using ImageJ software. The signal to noise ratio for each sample was calculated to allow comparison amongst the samples.

### Statistical analysis

The findings were analysed using the statistical software GraphPad Prism 5. The intensity of staining (CTCF) and signal to noise ratio was quantified as mean ± SEM. Unpaired t test was used to compare the means of CTCF between different concentrations of TNF-α in each time point. One way analysis of variance (ANOVA) test was used to perform multiple comparisons of mean CTCF at different concentrations of TNF-α and incubation periods. P values less than 0.05 were taken as being statistically significant (*P < 0.05, **P < 0.01, ***P < 0.001).

## Results

### Increased levels of P-selectin and VCAM-1 in activated mAEC

**Fig. 3:**
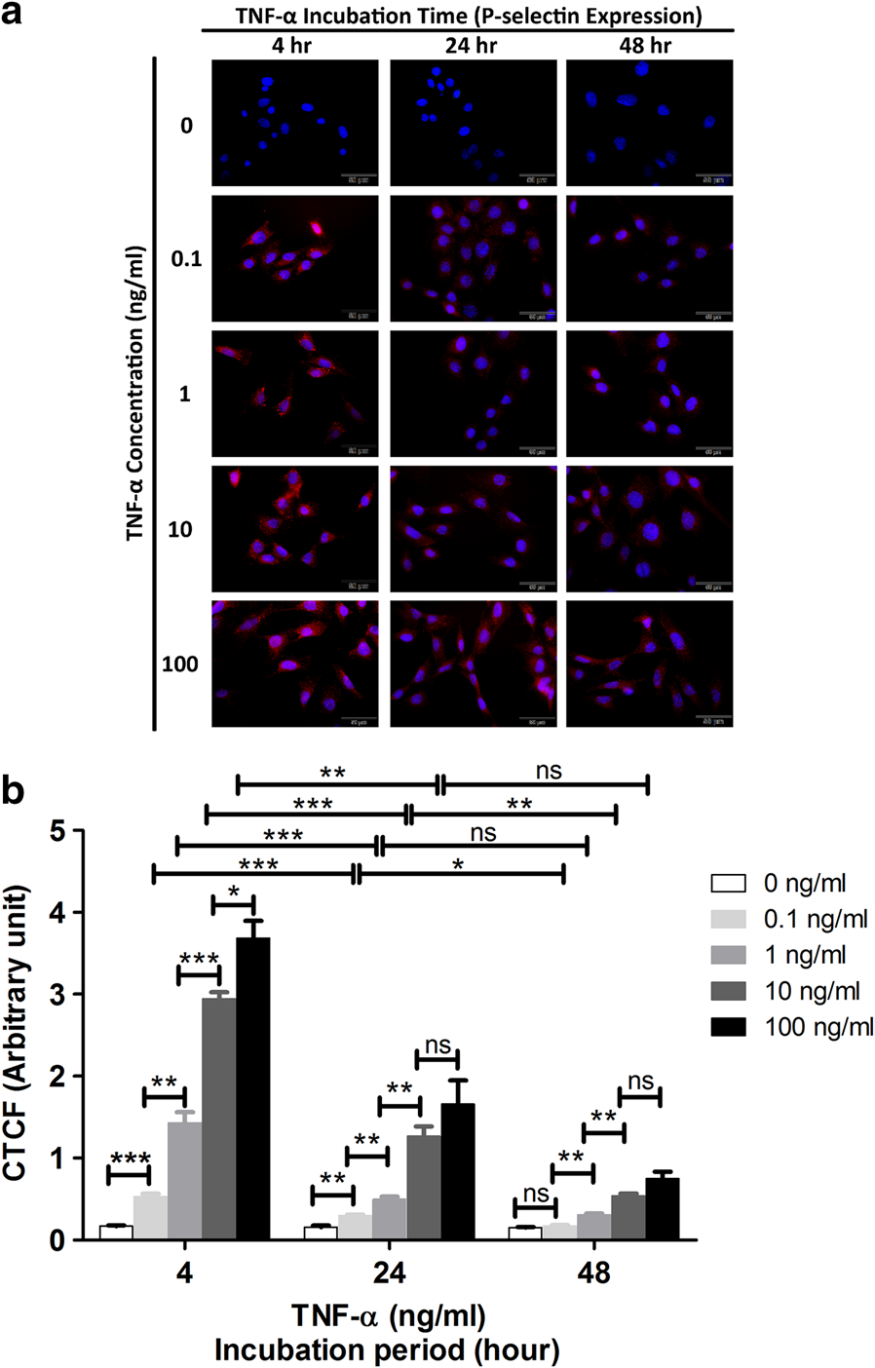
Immunocytochemistry for P-selectin on activated mAEC by different concentrations of TNF-α (0.1, 1, 10, 100 ng/mL) at different incubation period (4, 24, 48 h). **a** *Red* fluorescence signal for P-selectin expression. *Blue* DAPI staining for cell nuclei. Magnification ×60. **b** The intensity of staining obtained with anti-P-selectin antibody was quantified as corrected total cell fluorescence (CTCF) (mean ± SEM). Statistical significance is established at P < 0.05 (*P < 0.05, **P < 0.01, ***P < 0.001) and ns represents not significant

**Fig. 4:**
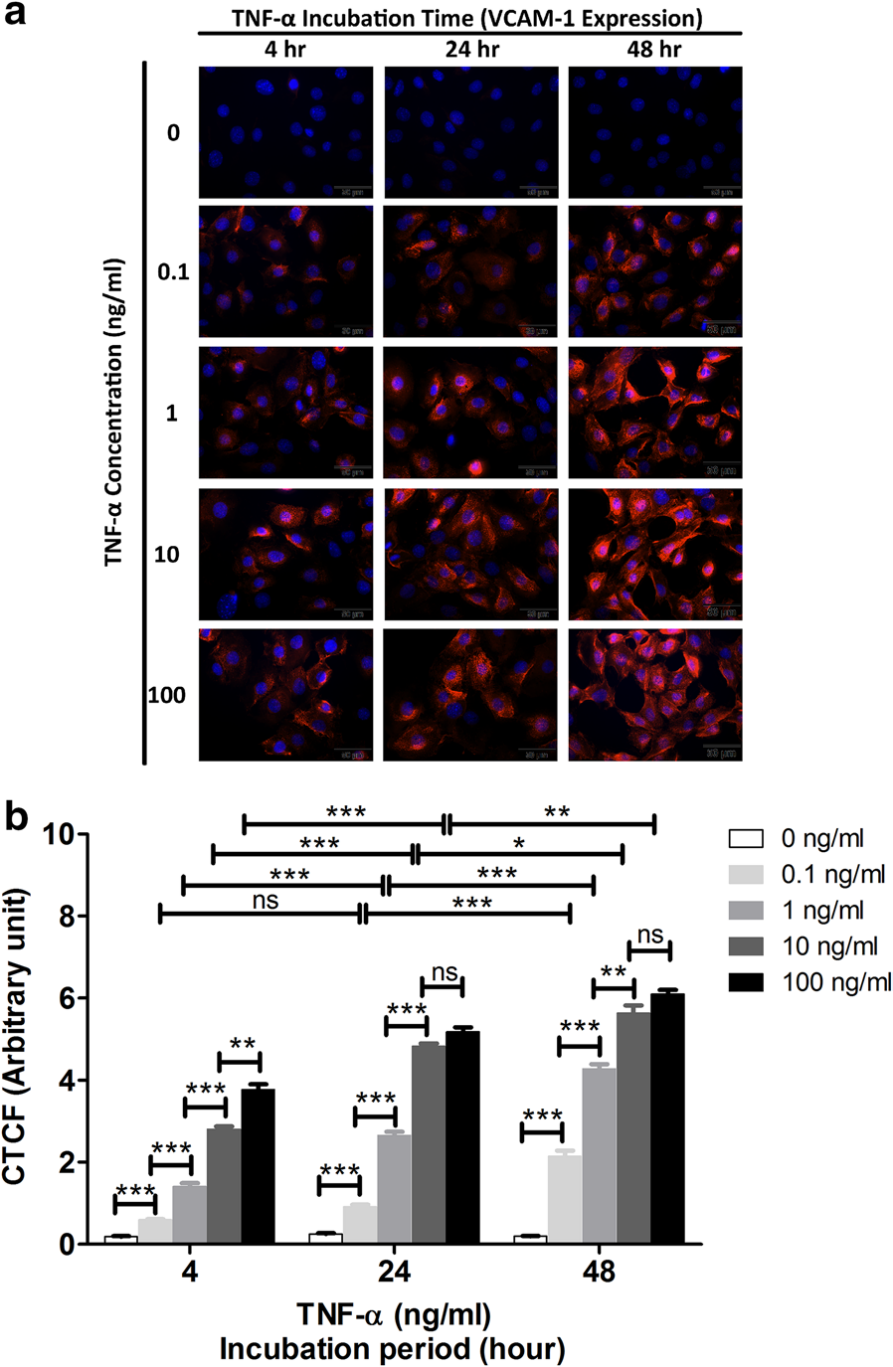
Immunocytochemistry for VCAM-1 on activated mAEC by different concentrations of TNF-α (0.1, 1, 10, 100 ng/mL) at different incubation period (4, 24, 48 h). **a** *Red* fluorescence signal for VCAM-1 expression. *Blue* DAPI staining for cell nuclei. Magnification ×60. **b** The intensity of staining obtained with anti-VCAM-1 antibody was quantified as corrected total cell fluorescence (CTCF) (mean ± SEM). Statistical significance is established at P < 0.05 (*P < 0.05, **P < 0.01, ***P < 0.001) and *ns* represents not significant

P-selectin and VCAM-1 were only detected in activated mAEC as shown by the red fluorescence signal at various concentrations of TNF-α and incubation periods, whilst no expression was observed in the untreated control cells (Figs. 3a, 4a). The dose response of TNF-α in mAEC was similar as detected by P-selectin and VCAM-1. Higher TNF-α concentration gave higher expression levels of both inflammatory markers, although the expression level was more obvious in VCAM-1 compared to P-selectin (Figs. 3b, 4b). In addition, VCAM-1 expression increased with time, with maximal signal at 48 h (Fig. 4b), while P-selectin level was maximal at 4 h post-TNF-α stimulation and then declined at 24 and 48 h (Fig. 3b).

### MRI signal concurs with the degree of inflammation in mAEC

**Fig. 5:**
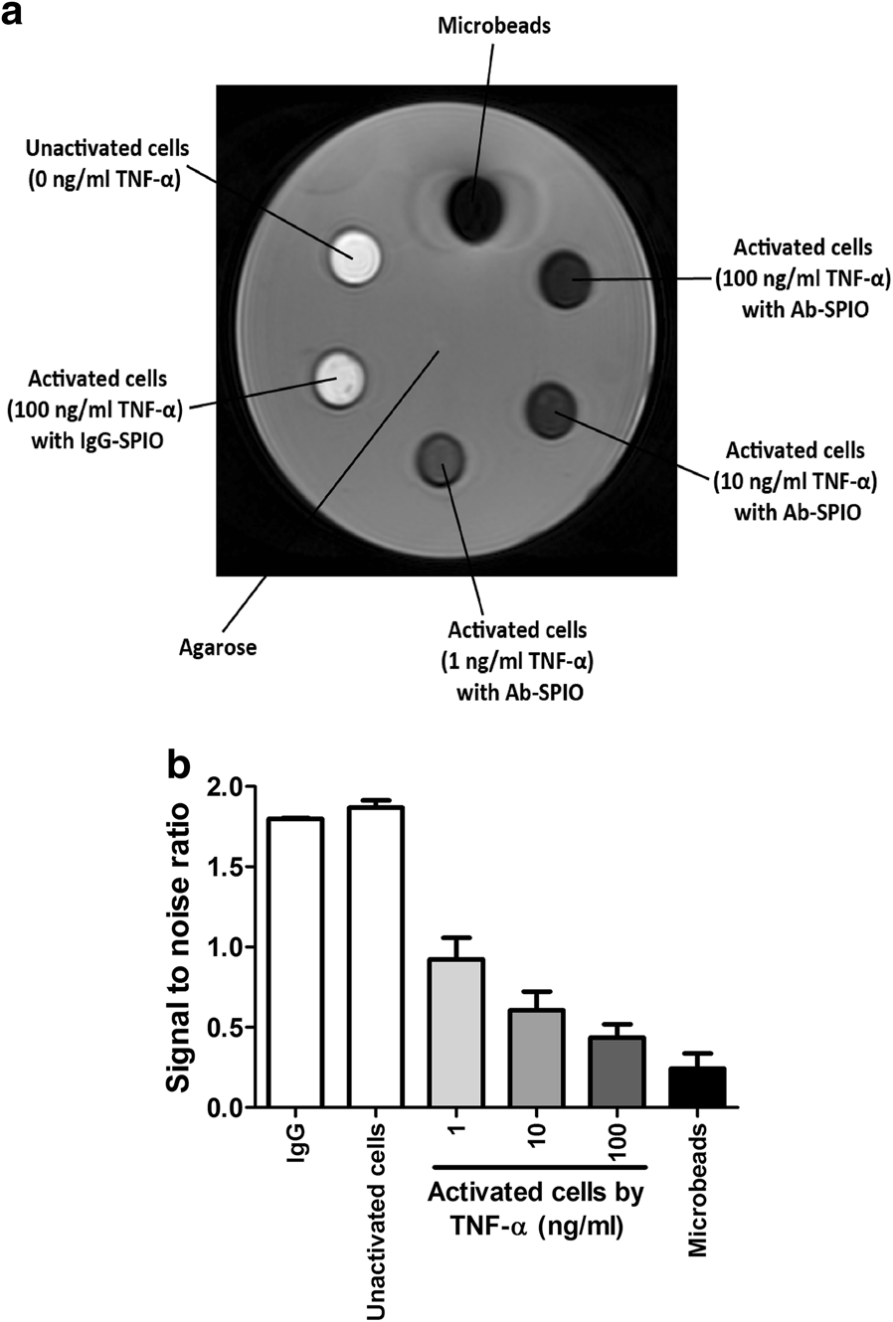
The transaxial view of cell phantom MRI. **a** T2 sequence. In clockwise direction: the microbeads, activated mAEC stimulated by TNF-α of different concentrations (100, 10 and 1 ng/mL), activated mAEC added with biotinylated IgG antibodies and microbeads and unactivated mAEC. **b** The S/N in streptavidin microbeads, positive control, was the smallest amongst all the samples. The activated cells stimulated by higher concentration of TNF-α produced a lower S/N. By contrast, the negative controls: (1) unactivated mAEC, and (2) activated mAEC added with biotinylated IgG antibodies and microbeads produced high S/N

The transaxial view of cell phantom MRI is illustrated in Fig. 5a. The streptavidin microbeads control were detected as signal void in T2 sequence by MRI and hence, appeared to be darkest. The activated mAEC stimulated by higher concentration of TNF-α were observed to produce greater signal void, and appeared darker on the MR image. The mAEC stimulated by 100 ng/mL TNF-α were observed to be the darkest amongst the three concentrations used, followed by 10 ng/mL, and finally the least with 1 ng/mL TNF-α. In contrast, the negative controls: (1) PBS (2) untreated mAEC, and (3) activated mAEC added with biotinylated IgG antibodies and microbeads were observed to produce minimal signal void, and appeared to be bright on the MR image.

In order to quantify these observations, the mean of the signal to noise ratio (S/N) in each sample in 5 consecutive MRI slices was measured. The results were illustrated in Fig. 5b. The S/N in microbeads control was 0.243, the lowest amongst all the samples. This was consistent with the observation that the microbeads control appeared to be the darkest amongst all samples. The activated cells stimulated by higher concentration of TNF-α produced a lower S/N.

## Discussion

### Activated mAEC

P-selectin is an adhesion molecule expressed on the luminal surface of vascular endothelial cells under inflammation [10]. It is not expressed by resting endothelial cells, but can be induced in vitro and in vivo following stimulation by pro-inflammatory mediators such as TNF-α or bacterial lipopolysaccharides [21, 22]. Previous studies have demonstrated P-selectin up-regulation in chronic inflammatory conditions such as rheumatoid arthritis [23] and inflammatory bowel disease [24]. By using P-selectin conjugated USPIO, Jacobin–Valat et al. demonstrated the expression of P-selectin on human activated platelets involved in the early stages of atherosclerosis [11]. Consistent with these studies, we confirmed that P-selectin expression was detected only on the activated mAECs, but not on the quiescent control mAEC (Fig. 3).

In our study, the P-selectin expression on activated mAEC was maximal at 4 h post-TNF-α stimulation, but declined after 24 and 48 h. Similar transient expression of P-selectin in vitro has been reported on murine endothelial cells with maximal expression at 3 h after stimulation with TNF-α [21]. We have also demonstrated that the level of P-selectin expression was dose-dependent on exposure to TNF-α, which is similar to E-selectin [25].

The role of VCAM-1 in the inflammatory initiation of atherosclerosis is well established in animal models [9]. In particular, VCAM-1 mediates firm adhesion, tethering, rolling of monocytes and facilitates their transendothelial migration at the developing atheroma [26]. Its expression is upregulated on activated endothelium under inflammatory conditions, including atherosclerosis [7], transplant rejection [27] and cancer [28]. Davies et al. [29] found VCAM-1 expression in a majority of post-mortem human coronary atherosclerotic plaques. Furthermore, decreased VCAM-1 expression in experimental atherosclerosis appears after treatment with either HMG-CoA reductase inhibitors [30] or angiotensin receptor blockers [31]. A reduction in atherosclerotic lesion formation was found in genetically modified mice with impaired VCAM-1 function [26].

VCAM-1 expression is not constitutive of, but does appear on endothelial cell surface in atheroprone sites even before the onset of visible disease, with persistent expression in more advanced lesions [4]. Consistent with these previous studies, we confirmed that VCAM-1 expression was only detected on the activated mAEC (Fig. 4), but not on the silent control cells (Fig. 4).

McAteer et al. [4] demonstrated a dose dependent effect on the in vitro binding of anti-VCAM-1 microparticles of iron-oxide (MPIO) to TNF-α-stimulated endothelial cells. We also observed similar dose-dependent increase in VCAM-1 expression, i.e. greater expression on the cells activated by higher concentration of TNF-α.

Unlike P-selectin, previous studies reported that VCAM-1 had different kinetics of expression on human endothelial cells, with a later onset and a more prolonged duration of at least 72 h compared to that of P-selectin [32]. Our study revealed similar results: greater level of VCAM-1 expression was observed with longer incubation period. The VCAM-1 expression on activated mAEC revealed at 4 h after stimulation by TNF-α, increased with longer incubation period and sustained a marked level of expression at 48 h.

The strict temporal regulation of these inducible endothelial adhesion molecules, their critical functions in atherosclerosis and their immediate accessibility via the circulation provide valuable targets for functional molecular imaging and targeted therapeutics. This potentially provides a new biologically based imaging modality beyond anatomy to identify the ‘at risk’ group with unstable plaque disease, affording the opportunity for early stroke preventive treatment.

### In vitro studies-MRI of cells

Figure 5 shows that the activated cells stimulated by higher concentration of TNF-α produced greater signal void and appeared darker in the MRI. The S/N values were consistent with these observations of MRI of the cell phantoms. The higher concentration of TNF-α used to activate the mAEC, the greater level of P-selectin and VCAM-1 expression on these cells. Therefore, greater amount of streptavidin microbeads were bound to the biotinylated anti-P-selectin and anti-VCAM-1 antibodies. Hence, greater signal loss was detected by MRI and lower S/N in mAEC activated by higher concentration of TNF-α. Therefore, we have successfully demonstrated an in vitro model to detect and characterise inflammation of endothelial cells by immunocytochemistry and MRI.

Similar in vitro studies with MRI of cell phantoms have been reported previously by McAteer et al. [4]. VCAM-1 conjugated microparticles of iron-oxide (MPIO) bound to TNF-α stimulated endothelial cells, but did not bind to unstimulated cells. The number of cell-bound VCAM–MPIO increased with increasing dose of TNF-α. MRI of cell phantoms showed punctate dark signal areas (hypointensity) representing VCAM-1–MPIO binding to cells, which increased with higher dose of TNF-α. Reynolds et al. demonstrated that E-selectin expression on activated vascular endothelium could be selectively and directly imaged non-invasively with in vivo MR by using anti-E-selectin antibody conjugated ultrasmall superparamagnetic iron oxide (USPIO) [15].

### Present challenges for in vivo molecular MRI

The present challenge for in vivo molecular imaging is to deliver an effective contrast agent in sufficient quantity to the arterial wall in vivo. For a contrast agent to be effective, it must provide high sensitivity and relaxivity for detection by MRI. It also needs to be stable under physiological conditions. In addition, it has to be membrane permeable, non-cytotoxic, functionalisable and highly specific. There is a rapid growth in the use of iron-oxide particles for detecting vascular inflammation in vivo with MRI [15, 33–35]. Tang et al. [33] and Kawahara et al. [34] utilised the non-specific uptake of USPIO and SPIO by macrophages to identify plaque inflammation in humans. It has been speculated that this contrast medium is phagocytosed by macrophages and accumulates in the atherosclerotic lesions. This was an indirect method to visualise the plaque by macrophage infiltration. A relatively high dose of the agent is necessary to obtain good contrast effect and accumulation takes up to 24 h. In contrast, our study is a pilot qualitative study where MRI with antibody-conjugated SPIO is used to assess the specific molecular events of vascular inflammation. Thus far, we have successfully developed an in vitro model to detect and characterise inflammation on the endothelial cells by both immunocytochemistry and MRI.

## Conclusion

We have established a robust in vitro model of vascular inflammation using mAEC induced with TNF-α. We have successfully detected and characterised the inflammation of endothelial cells by immunocytochemistry and MRI, which offers the potential for future translational studies to detect vascular inflammation in atherosclerosis. The results revealed a temporal expression in P-selectin and VCAM-1. In addition, the results suggested that the level of P-selectin and VCAM-1 expression was dose dependent on exposure to TNF-α.

This proof-of-concept work may form the basis for further translational studies to provide clinicians with a novel tool for in vivo assessment of atherosclerosis. This will potentially fulfil the current gap to identify vulnerable patients, to better define cardiovascular risk and to guide therapy more rationally, thereby contributing to a personalised approach to the management of atherosclerotic disease in the future.

## Abbreviations

mAEC: mouse aortic endothelial cells
CTA: computed tomography angiography
DAPI: 4′,6-diamidino-2-phenylindole
EDTA: ethylene diamine tetra acetic acid
FITC: fluorescein isothiocyanate
Ig: immunoglobulin
IL-1: interleukin-1
MPIO: microparticle of iron oxide
MRA: magnetic resonance angiography
MRI: magnetic resonance imaging
PBS: phosphate buffered saline
S/N: signal to noise ratio
T1: spin–lattice relaxation time
T2: spin–spin relaxation time
TNF-α: tumour necrosis factor-α
SPIO: superparamagnetic particles of iron oxide
VCAM-1: vascular cell adhesion molecule-1
